# Transition of Patients with Opioid Use Disorder from Buprenorphine to Extended‐Release Naltrexone: A Randomized Clinical Trial Assessing Two Transition Regimens

**DOI:** 10.1111/ajad.13024

**Published:** 2020-04-04

**Authors:** Sandra D. Comer, Paolo Mannelli, Danesh Alam, Antoine Douaihy, Narinder Nangia, Sarah C. Akerman, Abigail Zavod, Bernard L. Silverman, Maria A. Sullivan

**Affiliations:** ^1^ Department of Psychiatry Columbia University Irving Medical Center New York New York; ^2^ Department of Psychiatry and Behavioral Sciences Duke University Medical Center Durham North Carolina; ^3^ Northwestern Medicine Central DuPage Hospital Winfield Illinois; ^4^ Departments of Psychiatry and Medicine, Western Psychiatric Hospital University of Pittsburgh School of Medicine Pittsburgh Pennsylvania; ^5^ Alkermes, Inc. Waltham Massachusetts

## Abstract

**Background and Objective:**

When patients seek to discontinue buprenorphine (BUP) treatment, monthly injectable extended‐release naltrexone (XR‐NTX) may help them avoid relapse. The efficacy of low ascending doses of oral NTX vs placebo for patients transitioning from BUP to XR‐NTX is evaluated in this study.

**Methods:**

In a phase 3, hybrid residential/outpatient study, clinically stable participants with opioid use disorder (N = 101), receiving BUP for more than or equal to 3 months and seeking antagonist treatment, were randomized (1:1) to 7 residential days of descending doses of BUP and low ascending doses of oral NTX (NTX/BUP, n = 50) or placebo (PBO‐N/BUP, n = 51). Both groups received standing ancillary medications and psychoeducational counseling. Following negative naloxone challenge, participants received XR‐NTX (day 8). The primary endpoint was the proportion of participants who received and tolerated XR‐NTX.

**Results:**

There was no statistical difference between groups for participants receiving a first dose of XR‐NTX: 68.6% (NTX/BUP) vs 76.0% (PBO‐N/BUP; *P* = .407). The mean number of days with peak Clinical Opiate Withdrawal Scale (COWS) score less than or equal to 12 during the treatment period (days 1‐7) was similar for NTX/BUP and PBO‐N/BUP groups (5.8 vs 6.3; *P* = .511). Opioid withdrawal symptoms during XR‐NTX induction and post‐XR‐NTX observation period (days 8‐11) were mild and similar between groups (mean peak COWS score: NTX/BUP, 5.1 vs PBO‐N/BUP, 5.4; *P* = .464). Adverse events were mostly mild/moderate.

**Conclusions and Scientific Significance:**

Low ascending doses of oral NTX did not increase induction rates onto XR‐NTX compared with placebo. The overall rate of successful induction across treatment groups supports a brief BUP taper with standing ancillary medications as a well‐tolerated approach for patients seeking transition from BUP to XR‐NTX. (Am J Addict 2020;00:00–00)

## INTRODUCTION

Pharmacological treatment is the cornerstone of management for many patients with opioid use disorder (OUD).^1^ For individuals on buprenorphine (BUP), treatment needs and medication preference may change over time,^2^ and some patients may wish to discontinue BUP. However, relapse rates after BUP discontinuation have been found to range from 51% to 82% within the first 4 weeks,^3^, ^4^ with relapse rates as high as 90% after re‐stabilization and a second attempt at BUP discontinuation.^5^ Given this high relapse rate, the Substance Abuse and Mental Health Services Administration (SAMHSA; US Department of Health and Human Services) recommends that patients seeking to taper off agonist medication should be counseled about this risk, monitored during and after dose taper, and offered naltrexone extended‐release injectable suspension (XR‐NTX).^6^

Various regimens have been proposed to minimize the severity of withdrawal symptoms,^7^, ^8^ but no clear guidelines are available for the exact methods or the appropriate duration of this taper. Many practitioners offer patients seeking BUP discontinuation a slow descending taper of BUP over the course of many weeks or months. These patients often have trouble tapering below a BUP dose of 1 mg/d due to withdrawal discomfort and craving,^9^ especially if they are exposed to drug‐related environmental stimuli that can elicit craving.^10^ Such craving may interrupt the BUP taper, resulting in the need to increase the dose and extend the taper for a longer period. A recent 12‐year retrospective cohort study of adults treated with BUP in a large, urban primary care practice reported that although many patients desire to taper off BUP, only 48 of 1308 patients completed a taper (most without medical supervision), and more than half who discontinued had returned to BUP treatment within 2 years.^11^ Similarly, a recent study of adults in an outpatient primary care BUP program found that 85.5% of patients reported eventually wanting to discontinue BUP, but fewer than 10% were actively tapering; barriers included worry about withdrawal symptoms and fear of opioid relapse.^12^ Moreover, for patients who cease any pharmacological treatment for OUD, there is an associated increased risk of relapse and overdose events, including deaths.^13^, ^14^, ^15^

The opioid receptor antagonist XR‐NTX is indicated for the prevention of relapse to opioid dependence following opioid detoxification.^16^ When compared with those receiving placebo or treatment as usual, patients receiving XR‐NTX are more likely to remain in treatment, less likely to relapse to illicit opioid use, and have less craving for opioids in outpatient and in short‐ and long‐term inpatient settings.^17^, ^18^, ^19^, ^20^, ^21^ Recent randomized controlled trials have demonstrated that BUP and XR‐NTX have comparative effectiveness in relapse prevention and craving reduction once medication is initiated.^22^, ^23^ For patients wishing to transition from BUP to XR‐NTX, it is critically important to determine the method of transition that maximizes the likelihood of successful induction while minimizing withdrawal symptoms and the risk of potential relapse. Before initiating treatment with XR‐NTX, patients are recommended to be opioid‐free for a minimum of 7 to 10 days to avoid precipitating opioid withdrawal. Without active, medically supervised withdrawal management, patients may find this period difficult, and because many are likely to experience craving and withdrawal symptoms, their risk of relapse is increased and their induction onto XR‐NTX may be hindered. Various medically supervised withdrawal management regimens have been studied, including agonist tapers and the use of ancillary medications for symptom management.^7^ A number of studies have demonstrated the safety and feasibility of low ascending doses of oral NTX in combination with BUP and standing ancillary medications as a regimen for opioid withdrawal management and XR‐NTX induction, circumventing the need for a period of abstinence after an agonist taper.^17^, ^20^, ^24^, ^25^, ^26^ This combination of agonist‐antagonist provides transitory treatment with BUP and progressively introduces an opioid blockade with oral NTX. This current study builds upon a prior investigation,^24^ which examined the use of oral NTX, BUP, and standing ancillary medications to support transition from opioid agonists to XR‐NTX, to examine the use of this regimen in a different population. To the best of the authors’ knowledge, no randomized controlled studies have examined the comparative efficacy of regimens in patients seeking BUP discontinuation.

The development of a strategy to assist transition of BUP‐treated patients to antagonist therapy has immediate clinical relevance. Such a regimen would offer BUP‐treated patients an additional treatment option and encourage ongoing pharmacological support after BUP discontinuation, a time of high relapse risk. This study was designed to examine whether a regimen of low ascending doses of oral NTX in conjunction with BUP and standing ancillary medications would facilitate transition from BUP to XR‐NTX in a hybrid residential/outpatient setting. The present study represents the first systematic comparison of regimens for the transition of stable BUP‐treated patients to XR‐NTX.

## METHODS

### Study Design

**Figure 1:**
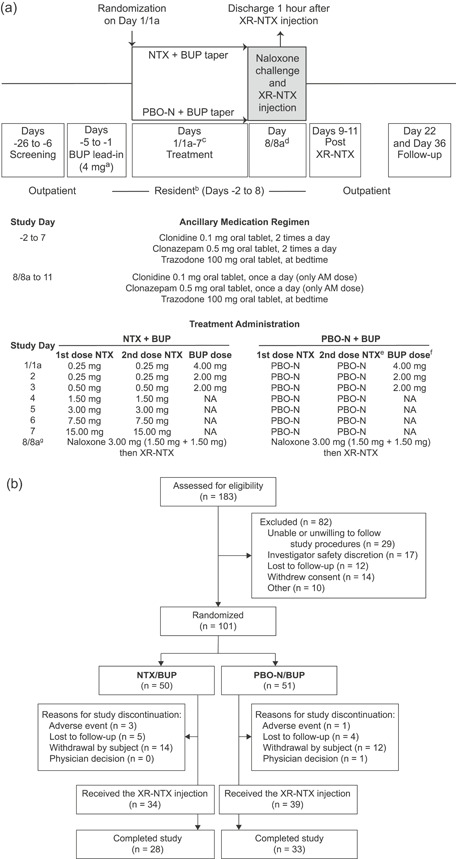
(a) Study design and (b) participant flow diagram. ^a^Participants maintained on less than 4 mg BUP at day‐5 continued their current dose until the treatment period taper called for further decrease. ^b^There was an option for earlier admission at the study clinician's discretion. ^c^Participants who did not qualify for randomization on day‐1 received day 1 BUP dose and repeated day 1 assessments and procedures the next day (day 1a). ^d^Participants who did not qualify to receive XR‐NTX on day 8 received day 7 study drug (NTX/PBO‐N) and completed day 7 assessments and procedures. day 8 assessments and procedures were repeated the next day (day 8a). ^e^The second dose of NTX/PBO‐N was administered at least 60 minutes after the first dose of NTX/PBO‐N after a clinical evaluation of tolerability (based on withdrawal symptoms). ^f^On days when participants received both BUP and NTX/PBO‐N, BUP was given sublingually immediately after the second dose of NTX/PBO‐N. ^g^If a participants did not qualify for the second dose of NTX/PBO‐N, then they still received the BUP dose for that day and were monitored in the clinic for 60 minutes after the BUP dose. If a participants had a positive naloxone challenge on day 8, they did not receive the XR‐NTX injection, but received day 7 study drug (NTX/PBO‐N) and completed day 7 assessments and procedures. They were offered the opportunity to remain in the residential unit overnight and they repeated the naloxone challenge the next day (day 8a) to qualify for XR‐NTX administration. Very few participants required a day 1a visit (n = 0) or 8a visit (n = 4). The two treatment arms had comparable number of participants admitted early (day‐5, day‐4, or day‐3 instead of day‐2) to begin the standing ancillary regimen (PBO‐N/BUP, n = 22; NTX/BUP, n = 20). BUP = buprenorphine; NA = not applicable; NTX = oral naltrexone; PBO‐N = placebo for oral naltrexone; XR‐NTX = extended‐release naltrexone.

This phase 3, multicenter, double‐blind, placebo‐controlled, parallel group, randomized study compared NTX + BUP or placebo‐NTX (PBO‐N) + BUP for induction onto XR‐NTX (VIVITROL; Alkermes, Inc; ClinicalTrials.gov NCT02696434) (Fig. 1a). All participants were administered standing ancillary medications, given that previous studies^8^ have shown that they assist with withdrawal symptoms and withholding such medications from one or more arms would be considered unethical.

The study was conducted at 10 sites in the United States from May 2016 to November 2017 (study completion) in accordance with the Declaration of Helsinki and Good Clinical Practice principles. The protocol, amendments, and informed consent were approved by a qualified institutional review board for each site, and all participants completed written informed consent before study participation.

### Participant Population

Participants aged 18 to 60 years, voluntarily seeking treatment for OUD, who expressed interest in transitioning to antagonist treatment with XR‐NTX were eligible if they (a) had a history of OUD for at least prior 6 consecutive months, as diagnosed by *Diagnostic and Statistical Manual of Mental Disorders* (5th ed.)^27^; (b) had a history of prescribed BUP (or BUP/naloxone) maintenance for prior 3 or more consecutive months and were currently BUP‐maintained; and (c) had been maintained on a daily BUP dose of less than or equal to 8 mg for at least 30 days before initiation of the BUP lead‐in period. Key exclusion criteria included the following: a positive urine drug screen for methadone, opiates (other than BUP) or oxycodone at screening on day‐5 (initiation of BUP lead‐in period); the use of NTX (oral or XR‐NTX) within 90 days before day‐5; the use of methadone within 30 days before day‐5; or a history of seizures or anticonvulsant therapy during the last 5 years.

### Study Procedures

#### Randomization

Participants were eligible if they were able to tolerate the lead‐in BUP dose, based on exhibiting minimal/mild opioid withdrawal symptoms (as confirmed by Clinical Opiate Withdrawal Scale [COWS] score less than or equal to 12). Participants were randomly assigned in a 1:1 ratio to one of two treatment groups: NTX + BUP or PBO‐N + BUP, stratified according to open‐label BUP dose (<8 vs 8 mg/d) at the time of initiation of the BUP lead‐in period.

For randomization to NTX or PBO‐N, a schedule was prepared by an independent biostatistician and uploaded into an Interactive Web Response System; the pharmacist contacted the system, which assigned the participant to a treatment group. The pharmacist was unblinded, and participants and investigators were blinded to treatment group assignment throughout the study.

#### Medically Supervised Withdrawal and Transition Protocol

The study included a BUP lead‐in period consisting of outpatient days −5 through −3 and residential days −2 and −1, and a treatment period consisting of transitional dosing with low ascending oral NTX or PBO‐N in conjunction with the BUP taper (residential days 1‐7). Up to 4 mg BUP was administered sublingually once daily for 5 days to establish a consistent daily dose before transitional dosing with oral NTX. After 5 days of stabilization on less than or equal to 4 mg BUP, on the morning of day 1 of the treatment period, participants underwent an assessment of withdrawal symptoms (COWS). Randomization occurred before study drug dosing on day 1.

Participants were offered psychoeducational counseling sessions at each study visit. Counseling sessions were conducted by qualified clinical study staff, which consisted of a review of common withdrawal symptoms, instruction on correct medication usage, and psychoeducation on the importance of adherence to study and ancillary medications.

The treatment period consisted of tapering doses of BUP (on days 1/1a‐3) in conjunction with low ascending doses of oral NTX or PBO‐N (days 1/1a‐7) (Fig. 1a).

The XR‐NTX induction and post‐XR‐NTX observation periods included a naloxone challenge and administration of XR‐NTX on day 8 before discharge.^24^ Participants who were unable to receive XR‐NTX on day 8 received day 7 study drug (NTX/PBO‐N) and completed day 7 assessments and procedures. Day 8 assessments and procedures were repeated the next day (day 8a). The XR‐NTX injection was followed by post‐XR‐NTX outpatient monitoring (days 9‐11).

Standing ancillary medications (clonidine 0.1 mg per os [p.o.; oral administration] twice daily, trazodone 100 mg p.o. at bedtime, and clonazepam 0.5 mg p.o. twice daily) were provided on days −2 to +7 to manage withdrawal symptoms. If a participant was admitted for the residential stay early (on days −5, −4, or −3) to manage craving or withdrawal symptoms, the ancillary regimen was initiated as soon as the participant was admitted. Participants continued using ancillary medication after the XR‐NTX injection on a modified schedule on days 8/8a‐11.

Vital signs, withdrawal symptoms (COWS and SOWS), and craving (visual analog scale [VAS]) were evaluated daily throughout the BUP lead‐in period, treatment period, and XR‐NTX induction and post‐XR‐NTX observation periods. Participants who had completed the treatment period and the XR‐NTX induction and post‐XR‐NTX observation periods (days 1/1a‐11) returned to the clinic for two outpatient follow‐up visits that occurred 2 weeks (day 22 ± 3) and 4 weeks (day 36 ± 3) after the XR‐NTX injection.

Urine drug screens were performed at all study visits and urine specimens were tested for opioids, illicit substances, and other drugs of abuse (opiates, methadone, oxycodone, BUP, cocaine, amphetamine, methamphetamine, tetrahydrocannabinol, benzodiazepines, barbiturates, propoxyphene, and phencyclidine). If a urine specimen tested positive for substances other than opioids, the participant was allowed to continue study participation unless the substance resulted in a serious adverse event (SAE) or raised a safety concern.

### Study Endpoints

#### Primary Endpoint

The primary efficacy endpoint was defined as the proportion of participants who received and tolerated an XR‐NTX injection on day 8/8a, as demonstrated by mild opioid withdrawal symptoms after XR‐NTX administration (COWS score of less than or equal to 12 or SOWS score of less than or equal to 10).

#### Secondary Endpoints

Secondary efficacy endpoints included the proportion of days with COWS peak scores less than or equal to 12 during the treatment period before XR‐NTX injection (days 1/1a‐7); the proportion of post‐XR‐NTX days (days 9‐11) in which participants in each group demonstrated mild opioid withdrawal (COWS score less than or equal to 12); the mean peak COWS scores during the treatment period, XR‐NTX induction, and post‐XR‐NTX observation period (days 1/1a‐11); the area under the curve (AUC) for COWS scores during the treatment period, XR‐NTX induction, and post‐XR‐NTX observation period (days 1/1a‐11); and the mean VAS score for “desire for opioids” (craving) during the treatment period, XR‐NTX induction, and post‐XR‐NTX observation period (days 1/1a‐11). Additional post hoc endpoints included peak SOWS scores during the treatment period, during XR‐NTX induction, and during the post‐XR‐NTX observation period.

#### Safety Endpoints

Safety endpoints included the incidence of treatment‐emergent adverse events (TEAEs), SAEs, and adverse events leading to discontinuation. Adverse events were described as preferred terms and system organ class categories using the Medical Dictionary for Regulatory Activities (MedDRA; Version 19.1).

### Statistical Analysis

A sample size of approximately 46 participants per treatment group was estimated to provide at least 90% power to detect a statistically significant difference between the two treatment groups at 5% level of significance in a two‐sided test. The sample size was calculated with the assumption that the proportion of participants who received and tolerated XR‐NTX was 90% in the NTX/BUP group and 60% in the PBO‐N/BUP group.

The safety population was defined as all randomized participants who received at least 1 dose of oral NTX or PBO‐N. All efficacy analyses were based on the safety population. Values are listed as mean (SD) except where indicated. The primary efficacy endpoint was analyzed for the safety population, using a logistic regression model that included treatment assignment and stratified randomization of BUP dose (<8 vs 8 mg/d) as factors. The analysis of the proportion of days with COWS peak scores less than or equal to 12 in each of the treatment period before XR‐NTX injection and in days 9‐11 (prespecified secondary endpoints) was carried out using a negative binomial model with treatment and stratified randomization of BUP dose (<8 vs 8 mg/d) as factors. Other prespecified secondary endpoints (mean VAS, AUC COWS) were examined using an analysis of covariance, with treatment and stratified randomization of BUP dose (<8 vs 8 mg/d) as factors and the corresponding baseline value as a covariate. Descriptive statistics are provided for other variables.

## RESULTS

### Participant Disposition

The safety population contained 101 participants, of whom 50 were randomized to the NTX/BUP group and 51 were randomized to the PBO‐N/BUP group. One participant was assigned to the NTX/BUP group, but he/she received PBO‐N/BUP. This participant's planned treatment (NTX/BUP) was used for all efficacy analyses (as per intention‐to‐treat) and actual treatment (PBO‐N/BUP) for all safety analyses. Demographic and baseline characteristics were similar between treatment groups (Table 1).

**Table 1:** Baseline participant characteristics

	NTX/BUP (n = 50)	PBO‐N/BUP (n = 51)	Total (N = 101)^a^
Age, median (range), y	35.0 (20‐57)	33.0 (23‐57)	34.0 (20‐57)
Male sex, n (%)	36 (72)	35 (69)	71 (70.3)
Race, n (%)			
White	46 (92)	47 (92)	93 (92.1)
Black or African‐American	4 (8)	3 (6)	7 (6.9)
Ethnicity, n (%)			
Hispanic or Latino	2 (4)	3 (6)	5 (5.0)
BMI, median (range), kg/m^2^	25.7 (18.8‐38.5)	25.3 (18.6‐39.2)	25.5 (18.6‐39.2)
COWS score, median (range)	3.0 (0‐15)	2.5 (0‐11)	3.0 (0‐15)
SOWS score, median (range)	2.0 (0‐39)	2.0 (0‐47)	2.0 (0‐47)
VAS craving score, median (range)	1.0 (0‐100)	1.0 (0‐80)	1.0 (0‐100)
HAM‐D score, median (range)	3.0 (0‐11)	2.0 (0‐24)	3.0 (0‐24)
Duration of OUD, n (%)			
>6 months but <12 months	5 (10.0)	9 (17.7)	14 (13.9)
1‐2 years	8 (16.0)	5 (9.8)	13 (12.9)
2‐3 years	6 (12.0)	5 (9.8)	11 (10.9)
3‐5 years	11 (22.0)	9 (17.7)	20 (19.8)
>5 years	20 (40.0)	23 (45.1)	43 (42.6)
BUP dose, n (%)			
8 mg/d	30 (60)	31 (61)	61 (60.4)
<8 mg/d	20 (40)	20 (39)	40 (39.6)
Duration of current BUP treatment, n (%)			
>3 months but <6 months	6 (12)	6 (12)	12 (11.9)
6‐12 months	9 (18)	9 (18)	18 (17.8)
1‐2 years	13 (26)	17 (33)	30 (29.7)
2‐3 years	8 (16)	8 (16)	16 (15.8)
>3 years	14 (28)	11 (22)	25 (24.8)

BMI = body mass index; BUP = buprenorphine; COWS = Clinical Opiate Withdrawal Scale; HAM‐D = Hamilton Depression Rating Scale; NTX = oral naltrexone; OUD = opioid use disorder; PBO‐N = placebo for oral naltrexone; SOWS = Subjective Opiate Withdrawal Scale; VAS = visual analog scale.

^a^ One participant was assigned to the NTX/BUP group, but he/she received PBO‐N/BUP.

Sixty‐one (60.4%) participants completed the study (completed all visits during the follow‐up period), 23 (22.8%) participants discontinued the study during the treatment period, and 17 (16.8%) participants discontinued the study during the follow‐up period (Fig. 1b). Study completion and discontinuation were similar between treatment arms.

### Primary Endpoint

Both treatment arms showed comparable rates of induction onto XR‐NTX: 68.6% in the NTX/BUP group and 76.0% in the PBO‐N/BUP group (odds ratio [95% confidence interval], 0.68 [0.28, 1.68]; *P* = .407) (Table 2); therefore, the study did not demonstrate a statistically significant difference in treatment arms when evaluating the primary endpoint.

In a post hoc analysis of mean induction onto XR‐NTX by original BUP dose group, a numerically greater proportion of participants who were maintained on less than 8 mg BUP daily completed the transition, compared with participants who were maintained on the 8 mg dose (Table 2). For participants maintained on the less than 8 mg BUP dose, a higher proportion transitioned to XR‐NTX in the PBO‐N/BUP (95%) vs NTX/BUP treatment arm (75%). For participants maintained on the 8 mg dose, induction onto XR‐NTX was similar for both treatment arms (PBO‐N/BUP, 63%; NTX/BUP, 65%).

**Table 2:** Study endpoints

Mean (SD), unless stated	NTX/BUP (n = 51)	PBO‐N/BUP (n = 50)	*P* value
Received and tolerated XR‐NTX injection on day 8/8a, n (%)^a^	35 (69)	38 (76)	.407
Participants with BUP dose <8 mg/d, n/N in treatment arm (%)	15/20 (75)	19/20 (95)	
Participants with BUP dose 8 mg/d, n/N in treatment arm (%)	20/31 (65)	19/30 (63)	
Number of days with COWS peak score ≤12 during the treatment period (days 1/1a‐7)^b^	5.8 (1.6)	6.3 (1.4)	.511
Number of days with COWS peak score ≤12 during the post‐XR‐NTX observation period (days 9‐11)^b^	2.4 (0.9)	2.6 (0.8)	.716
Peak COWS score during the treatment period (days 1/1a‐7)	6.0 (3.7)	5.0 (2.8)	.151
Peak COWS score during the XR‐NTX induction and post‐XR‐NTX observation period (days 8/8a‐11)	5.1 (2.6)	5.4 (3.3)	.464
AUC for COWS score during the treatment period (days 1/1a‐8/8a)	4.5 (3.1)	3.9 (2.3)	.432
AUC for COWS score during the XR‐NTX induction and post‐XR‐NTX observation period (days 9‐11)	4.7 (2.8)	4.8 (3.1)	.727
Peak SOWS score during the treatment period (days 1/1a‐7)	11.2 (11.9)	7.5 (7.9)	⋯
Peak SOWS score during the XR‐NTX induction and post‐XR‐NTX observation period (days 8/8a‐11)	8.0 (11.4)	7.6 (8.5)	⋯
VAS craving score during the treatment period, XR‐NTX induction, and post‐XR‐NTX observation period (days 1/1a‐11)^b^	11.4 (16.2)	8.8 (12.2)	.088
VAS craving score during the treatment period (days 1/1a‐7)	12.1 (17.5)	8.7 (12.2)	.045
VAS craving score during XR‐NTX induction and post‐XR‐NTX observation period (days 8/8a‐11)	6.3 (12.6)	8.3 (14.4)	.578

AUC = area under the curve; BUP = buprenorphine; COWS = Clinical Opiate Withdrawal Scale; NTX = oral naltrexone; PBO‐N = placebo for oral naltrexone; SOWS = Subjective Opiate Withdrawal Scale; VAS = visual analog scale; XR‐NTX = extended‐release naltrexone.

^a^ XR‐NTX tolerability: after the XR‐NTX injection on day 8/8a, the participant's opioid withdrawal symptoms are mild (1‐hour post‐dose XR‐NTX COWS score ≤12 or SOWS score ≤10).

^b^ Predefined secondary study outcomes.

### Secondary and Additional Endpoints

**Figure 2:**
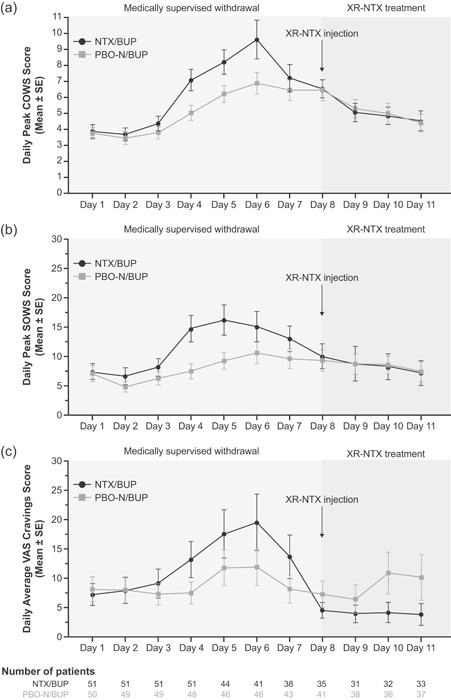
Secondary endpoints. (a) Daily peak COWS scores, (b) daily peak SOWS scores, (c) daily mean VAS craving scores and mean daily peak scores (COWS and SOWS), and mean daily average scores (VAS) are shown over the course of the medically supervised withdrawal period, XR‐NTX injection, and through the post‐XR‐NTX observation period. Error bars represent ±SEM. The number of patients assessed for each measure and time point is listed below the figure. The XR‐NTX induction and post‐XR‐NTX observation period included a naloxone challenge and administration of XR‐NTX on day 8 before discharge. BUP = buprenorphine; COWS = Clinical Opiate Withdrawal Scale; NTX = oral naltrexone; PBO‐N = placebo for oral naltrexone; SOWS = Subjective Opiate Withdrawal Scale; VAS = visual analog scale; XR‐NTX = extended‐release naltrexone.

All procedures were well tolerated, with COWS scores being considered mild across all treatment groups and days. The number of days with COWS peak scores less than or equal to 12 during the treatment period (days 1/1a‐7; *P* = .511) and during the post‐XR‐NTX injection observation period (days 9‐11; *P* = .716) was similar between the NTX/BUP and PBO‐N/BUP groups, as were mean peak COWS scores during the treatment period (days 1/1a‐7; *P* = .151) and during the XR‐NTX induction and post‐XR‐NTX observation period (days 8/8a‐11; *P* = .464) (Table 2). The mean AUCs for COWS scores were similar between the NTX/BUP and PBO‐N/BUP groups during the treatment period and the XR‐NTX induction and post‐XR‐NTX observation period (Table 2). Mean peak SOWS scores are presented in Table 2. Mean daily peak COWS scores and mean daily peak SOWS scores are presented in Fig. 2a and 2b, respectively.

The mean VAS craving scores for the NTX/BUP and PBO‐N/BUP groups during the treatment period (days 1/1a‐7) were 12.1 and 8.7, and during the XR‐NTX induction and post‐XR‐NTX observation period (days 8‐11), the scores were 6.3 and 8.3 (Table 2). Mean VAS craving scores during the treatment period, XR‐NTX induction, and post‐XR‐NTX observation period (days 1/1a‐11) were similar (Table 2). Mean daily VAS scores are presented in Fig. 2c.

### Safety

TEAEs were similar in type and frequency between the two treatment arms; they were reported in 76.0% (n = 38) of participants in the NTX/BUP group and 72.5% (n = 37) in the PBO‐N/BUP group (Table 3). The most common AEs were consistent with symptoms of mild‐to‐moderate opioid withdrawal. Among the TEAEs occurring at a rate of at least 10% among participants, rates of diarrhea, myalgia, and nausea were numerically greater in the NTX/BUP group compared with the PBO‐N/BUP group. By contrast, the reported rate of constipation was numerically greater in the PBO‐N/BUP group compared with the NTX/BUP group. One participant had two serious events, which included suicidal ideation and a psychotic disorder (“brief psychotic episode”), both deemed by the investigator to be probably not related to oral NTX or BUP, but possibly related to XR‐NTX. The three AEs that led to the discontinuation were myalgia, anxiety, and drug withdrawal syndrome. During the study, there was no occurrence of overdoses or deaths.

**Table 3:** Adverse events during treatment period

	NTX/BUP (n = 50)	PBO‐N/BUP (n = 51)	Total (N = 101)
Any TEAE, n (%)	38 (76)	37 (73)	75 (74.3)
Mild	18 (36)	22 (43)	40 (39.6)
Moderate	19 (38)	13 (26)	32 (31.7)
Severe	1 (2)	2 (4)	3 (3.0)
TEAEs experienced by ≥10% of participants, n (%)			
Anxiety	15 (30)	15 (29)	30 (29.7)
Insomnia	13 (26)	13 (26)	26 (25.7)
Diarrhea	16 (32)	9 (18)	25 (24.8)
Myalgia	10 (20)	6 (12)	16 (15.8)
Nausea	9 (18)	5 (10)	14 (13.9)
Abdominal pain, upper	7 (14)	6 (12)	13 (12.9)
Headache	5 (10)	3 (6)	8 (7.9)
Constipation	3 (6)	6 (12)	9 (8.9)
Any SAE, n (%)^a^			
Suicidal ideation	0	1 (2)	1 (1.0)
AEs leading to discontinuation, n (%)	3 (6)	0	3 (3.0)

AE = adverse event; BUP = buprenorphine; NTX = oral naltrexone; PBO‐N = placebo for oral naltrexone; SAE = serious adverse event; TEAE = treatment‐emergent adverse event; XR‐NTX = extended‐release naltrexone.

^a^ One participant had two SAEs, which included suicidal ideation (probably not related to oral NTX or BUP, but possibly related to XR‐NTX) and psychotic disorder (“brief psychotic episode,” probably not related to oral NTX or BUP, but possibly related to XR‐NTX). The event of psychotic disorder occurred in the follow‐up period.

## DISCUSSION

To the best of the authors’ knowledge, this was the first randomized controlled trial to evaluate two different regimens to transition BUP‐treated patients to XR‐NTX. Compared with placebo, the addition of low ascending doses of oral NTX to a BUP taper with standing ancillary medications did not improve XR‐NTX induction rates using this 7‐day hybrid residential/outpatient treatment protocol. Both treatment arms showed comparable rates of induction onto XR‐NTX. Furthermore, the treatment arm receiving oral naltrexone, compared with placebo, had greater rates of nausea, diarrhea, and myalgia, suggesting effects of precipitated withdrawal. The high levels of induction observed in the study (72% overall) support the use of a brief BUP taper in combination with a standing ancillary regimen and psychoeducational counseling for individuals voluntarily seeking transition from BUP to XR‐NTX, particularly for those on BUP doses less than 8 mg/d at the time of protocol initiation. The present findings support the use of readily available ancillary medications (clonidine, trazodone, and clonazepam) for transitioning patients seeking to discontinue BUP and initiate XR‐NTX.

Participants who entered the study on a lower (<8 mg) BUP dose and received PBO‐NTX/BUP had a 95% success rate of induction onto XR‐NTX (compared with a 63% success rate for subjects who entered the study on 8 mg BUP and received PBO‐NTX/BUP). This finding supports a clinical strategy of careful tapering of the BUP dose in patients seeking to transition from BUP to XR‐NTX, which is consistent with results from previous trials showing that lower severity of OUD is associated with a higher likelihood of successful XR‐NTX induction.^24^, ^26^ It is worth noting that although the rates of transition to XR‐NTX among participants in the higher‐dose BUP group were similar across the treatment arms (63% vs 65%), the lower‐dose BUP group had greater success in transitioning in the PBO‐NTX/BUP arm, compared to the NTX/BUP arm (95% vs 75%). These findings suggest that individuals on lower doses of BUP (<8 mg/d) are more likely to successfully transition to XR‐NTX using a regimen consisting of a BUP taper and ancillary medication, without the use of oral naltrexone. In addition, with the use of a combination of standing ancillary medications and a BUP taper, patients able to reduce their dose of BUP to less than 8 mg/d may be more likely to succeed in the transition to opioid antagonist therapy.

The combined outpatient and residential design of this trial was selected to ensure tolerability of lower‐dose BUP before introduction of the treatment regimen and to enable thorough assessment of opioid withdrawal during the transition from BUP to XR‐NTX. In addition, this design was chosen to minimize the risks of attrition before XR‐NTX induction, given that BUP discontinuation without subsequent pharmacologic support is associated with heightened risk of relapse and overdose. Overall, subjective and objective withdrawal symptoms and craving severity were mild to moderate. Both regimens were well tolerated by BUP‐treated participants inducted onto XR‐NTX. Most AEs were of mild‐to‐moderate severity and consistent with symptoms of opioid withdrawal.^24^ Administration of low‐dose oral NTX in a 7‐day ascending taper was associated initially with worsened withdrawal symptoms during transition, compared with placebo. However, reductions in craving scores were observed after induction, and there was no statistical difference in COWS/SOWS between treatment groups.

Previous studies have described the benefits of ascending oral NTX in 5‐ to 7‐day transition regimens by the introduction of a gradual opioid blockade.^20^, ^25^, ^26^, ^28^ Combining a brief BUP taper with low ascending doses of oral NTX before a first XR‐NTX injection has been postulated to reduce physiological dependence by providing a partial agonist while concurrently introducing a gradual opioid blockade.^20^, ^24^, ^25^, ^26^ A recent large placebo‐controlled study in patients with OUD transitioning to XR‐NTX in an outpatient setting over 7 days, found that standing doses of ancillary medication performed as well as transition regimens that included low ascending doses of oral NTX, with or without BUP.^24^ The present trial similarly demonstrated that low ascending doses of oral NTX did not increase the likelihood of receiving a first dose of XR‐NTX following this 7‐day transition regimen. Given these convergent recent findings, future research should aim to understand if there is a role for oral NTX in facilitating rapid (3‐ or 4‐day) XR‐NTX induction strategies.

The combination of a fixed‐dose ancillary regimen (including clonidine, clonazepam, and trazodone), a 7‐day transition period, and psychoeducational counseling represented a well‐tolerated approach to the management of opioid withdrawal symptoms, leading to XR‐NTX induction in a hybrid inpatient/outpatient setting. Currently available ancillary medications have immediate potential in clinical practice to benefit BUP‐maintained patients who are seeking to transition to XR‐NTX. In addition, the relatively high rate of successful induction onto XR‐NTX observed in this study, compared with prior published rates for transition from active OUD,^24^, ^26^ is consistent with enrollment of a stable patient population that had already demonstrated a commitment to medication for OUD and the use of an inpatient XR‐NTX induction.

The exclusion of patients with consistently positive urine drug screening for illicit or non‐prescribed opioids is one limitation of the study. Furthermore, results from randomized controlled trials and, particularly the use of a hybrid residential/outpatient setting, may not fully reflect real‐world practice. In addition, low doses of oral NTX are not currently approved for commercial use. Furthermore, this study was not designed to assess differences in “as needed” (PRN) use of ancillary medications; varying frequency of use in each treatment arm may have contributed to our findings.

Clinicians conducting XR‐NTX induction in an outpatient setting may rely upon their standard methods of assessing opioid withdrawal, craving, and opioid use. Although the use of a standing regimen of ancillary medications is supported by the current study, clinicians working in an outpatient setting may prefer a different combination of ancillary medications that they have found to be effective.

To the best of our knowledge, this is the first randomized study examining the efficacy of regimens to support the transition from BUP to XR‐NTX, for individuals seeking to discontinue BUP. Although the low ascending doses of oral NTX did not improve the rate of induction compared with placebo, the results support the feasibility of a brief BUP taper in combination with standing ancillary medications and psychoeducational counseling in a 7‐day regimen as a well‐tolerated approach for patients seeking transition from BUP to XR‐NTX in a residential setting. Future research is needed to explore the utility of this transition regimen in the outpatient practice setting.

*Medical writing support was provided by Tabasum Mughal, PhD (ApotheCom, UK), and Janelle Keys, PhD, CMPP (ProScribe, part of Envision Pharma Group), both funded by Alkermes, Inc*. *This study was sponsored by Alkermes, Inc, the manufacturer/licensee of XR‐NTX. Alkermes, Inc. was involved in the study design, data collection, data analysis, and preparation of the manuscript*.

*The authors thank the investigators and participants for their contribution to this study*.

## Declaration of Interest

SA, AZ, and MAS are employees and may be shareholders of Alkermes, Inc; NN and BLS are former employees of Alkermes, Inc. SDC and PM have received consultation fees and grants from Alkermes and other pharmaceutical companies. DA and AD have participated in advisory boards and received grants from Alkermes. MAS previously received study medication from Alkermes for a NIDA‐funded investigation.

## Author Contributions

All authors participated in the interpretation of study results, in the drafting, critical revision, and approval of the final version of the manuscript. SDC, PM, DA, and AD were study investigators; SDC was also involved in the study design. SA, AZ, BLS, MAS, and NN were involved in the study design and data analyses. NN conducted the statistical analyses.

## Data Sharing Statement

The data collected in this study are proprietary to Alkermes, Inc. Alkermes, Inc is committed to public sharing of data in accordance with applicable regulations and laws.

## References

[1] Douaihy AB , Kelly TM , Sullivan C . Medications for substance use disorders. Soc Work Pub Health. 2013;28:264‐278. 23731419 10.1080/19371918.2013.759031 PMC3767185

[2] Huhn AS , Tompkins DA , Dunn KE . The relationship between treatment accessibility and preference amongst out‐of‐treatment individuals who engage in non‐medical prescription opioid use. Drug Alcohol Depend. 2017;180:279‐285. 28942031 10.1016/j.drugalcdep.2017.08.019 PMC5648596

[3] Ling W , Hillhouse M , Domier C , et al. Buprenorphine tapering schedule and illicit opioid use. Addiction. 2009;104:256‐265. 19149822 10.1111/j.1360-0443.2008.02455.x PMC3150159

[4] Nielsen S , Hillhouse M , Thomas C , et al. A comparison of buprenorphine taper outcomes between prescription opioid and heroin users. J Addict Med. 2013;7:33‐38. 23222095 10.1097/ADM.0b013e318277e92e PMC3567310

[5] Weiss RD . Adjunctive counseling during brief and extended buprenorphine‐naloxone treatment for prescription opioid dependence: a 2‐phase randomized controlled trial. Arch Gen Psychiatry. 2011;68:1238‐1246. 22065255 10.1001/archgenpsychiatry.2011.121 PMC3470422

[6] Substance Abuse and Mental Health Services Administration . Medications for opioid use disorder. Treatment Improvement Protocol (TIP) Series 63, Executive Summary. HHS Publication No. (SMA) 18‐5063EXSUMM. Substance Abuse and Mental Health Services Administration, Rockville, MD; 2018.

[7] Bisaga A , Mannelli P , Sullivan MA , et al. Antagonists in the medical management of opioid use disorders: historical and existing treatment strategies. Am J Addict. 2018;27:177‐187. 29596725 10.1111/ajad.12711 PMC5900907

[8] Sigmon SC , Bisaga A , Nunes EV , et al. Opioid detoxification and naltrexone induction strategies: recommendations for clinical practice. Am J Drug Alcohol Abuse. 2012;38:187‐199. 22404717 10.3109/00952990.2011.653426 PMC4331107

[9] Northrup TF , Stotts AL , Green C , et al. Opioid withdrawal, craving, and use during and after outpatient buprenorphine stabilization and taper: a discrete survival and growth mixture model. Addict Behav. 2015;41:20‐28. 25282598 10.1016/j.addbeh.2014.09.021 PMC4252696

[10] Weiss F . Neurobiology of craving, conditioned reward and relapse. Current Opin Pharm. 2005;5:9‐19. 10.1016/j.coph.2004.11.001 15661620

[11] Weinstein ZM , Gryczynski G , Cheng DM , et al. Tapering off and returning to buprenorphine maintenance in a primary care office based addiction treatment (OBAT) program. Drug Alcohol Depend. 2018;189:166‐171. 29958128 10.1016/j.drugalcdep.2018.05.010 PMC6139651

[12] Stein MD , Conti MT , Herman DS , et al. Worries about discontinuing buprenorphine treatment: scale development and clinical correlates. Am J Addict. 2019;28:270‐276. 30993833 10.1111/ajad.12884 PMC6591066

[13] Digiusto E , Shakeshaft A , Ritter A , et al. Serious adverse events in the Australian National Evaluation of Pharmacotherapies for Opioid Dependence (NEPOD). Addiction. 2004;99:450‐460. 15049745 10.1111/j.1360-0443.2004.00654.x

[14] Sordo L , Barrio G , Bravo MJ , et al. Mortality risk during and after opioid substitution treatment: systematic review and meta‐analysis of cohort studies. BMJ. 2017;357:j1550. 28446428 10.1136/bmj.j1550 PMC5421454

[15] Williams AR , Samples H , Crystal S , et al. Acute care, prescription opioid use, and overdose following discontinuation of long‐term buprenorphine treatment for opioid use disorder. Am J Psychiatry. 2019;177:117‐124. 10.1176/appi.ajp.2019.19060612 31786933 PMC7002204

[16] Alkermes Inc . VIVITROL (naltrexone for extended‐release injectable suspension). Prescribing information. https://www.vivitrol.com/content/pdfs/prescribing‐information.pdf. Accessed July 20, 2019.

[17] Dakwar E , Kleber HD . Naltrexone‐facilitated buprenorphine discontinuation: a feasibility trial. J Subst Abuse Treat. 2015;53:60‐63. 25819700 10.1016/j.jsat.2015.01.004

[18] Herbeck DM , Jeter KE , Cousins SJ , et al. Gender differences in treatment and clinical characteristics among patients receiving extended release naltrexone. J Addict Dis. 2016;35:305‐314. 27192330 10.1080/10550887.2016.1189659

[19] Krupitsky E , Nunes EV , Ling W , et al. Injectable extended‐release naltrexone (XR‐NTX) for opioid dependence: long‐term safety and effectiveness. Addiction. 2013;108:1628‐1637. 23701526 10.1111/add.12208

[20] Mannelli P , Wu LT , Peindl KS , et al. Extended release naltrexone injection is performed in the majority of opioid dependent patients receiving outpatient induction: a very low dose naltrexone and buprenorphine open label trial. Drug Alcohol Depend. 2014;138:83‐88. 24602363 10.1016/j.drugalcdep.2014.02.002 PMC4017322

[21] Nunes EV , Gordon M , Friedmann PD , et al. Relapse to opioid use disorder after inpatient treatment: protective effect of injection naltrexone. J Subst Abuse Treat. 2017;85:49‐55. 28473233 10.1016/j.jsat.2017.04.016 PMC5755382

[22] Lee JD , Nunes EV Jr , Novo P , et al. Comparative effectiveness of extended‐release naltrexone versus buprenorphine‐naloxone for opioid relapse prevention (X:BOT): a multicentre, open‐label, randomised controlled trial. Lancet. 2018;391:309‐318. 29150198 10.1016/S0140-6736(17)32812-X PMC5806119

[23] Tanum L , Solli KK , Latif ZH , et al. Effectiveness of injectable extended‐release naltrexone vs daily buprenorphine‐naloxone for opioid dependence: a randomized clinical noninferiority trial. JAMA Psychiatry. 2017;74:1197‐1205. 29049469 10.1001/jamapsychiatry.2017.3206 PMC6583381

[24] Bisaga A , Mannelli P , Yu M , et al. Outpatient transition to extended‐release injectable naltrexone for patients with opioid use disorder: a phase 3 randomized trial. Drug Alcohol Depend. 2018;187:171‐178. 29674251 10.1016/j.drugalcdep.2018.02.023

[25] Sigmon SC , Dunn KE , Saulsgiver K , et al. A randomized, double‐blind evaluation of buprenorphine taper duration in primary prescription opioid abusers. JAMA Psychiatry. 2013;70:1347‐1354. 24153411 10.1001/jamapsychiatry.2013.2216 PMC4131728

[26] Sullivan M , Bisaga A , Pavlicova M , et al. Long‐acting injectable naltrexone induction: a randomized trial of outpatient opioid detoxification with naltrexone versus buprenorphine. Am J Psychiatry. 2017;174:459‐467. 28068780 10.1176/appi.ajp.2016.16050548 PMC5411308

[27] American Psychiatric Association . Diagnostic and Statistical Manual of Mental Disorders. 5th ed. Washington, DC: American Psychiatric Association; 2013.

[28] Sibai M , Mishlen K , Nunes E , et al. A five‐day outpatient induction onto XR‐naltrexone in patients with opioid use disorder. Poster presented at 80th Annual College on Problems of Drug Dependence Meeting, June 9‐14, 2018; San Diego, CA.

